# The importance of critically short telomere in myelodysplastic syndrome

**DOI:** 10.1186/s40364-022-00426-9

**Published:** 2022-11-10

**Authors:** Dong-Yeop Shin, Kyu Min Lim, Hee Sue Park, Sunghoon Kwon, Sung-Soo Yoon, Dong-Soon Lee

**Affiliations:** 1 Division of Hematology and Medical Oncology, Department of Internal Medicine, Seoul National University Hospital, 101 Daehag-ro, Seoul, Republic of Korea; 2 Center for Medical Innovation, Biomedical Research Institute, Seoul National University Hospital, Seoul, Republic of Korea; 3 Cancer Research Institute, Seoul National University College of Medicine, Seoul, Republic of Korea; 4 Department of Laboratory Medicine, Chungbuk National University Hospital, Cheongju, Republic of Korea; 5 Department of Electrical and Computer Engineering, Seoul National University, Seoul, Republic of Korea; 6 Interdisciplinary Program in Bioengineering, Seoul National University, Seoul, Republic of Korea; 7 Bio-MAX Institute, Seoul National University, Seoul, Republic of Korea; 8 Department of Laboratory Medicine, Seoul National University College of Medicine, Seoul National University Hospital, 101 Daehag-ro, Seoul, 03080 Republic of Korea

**Keywords:** TeSLA, Short telomere, Myelodysplastic syndrome

## Abstract

A few critically short telomeres trigger genomic instability regardless of average telomere length (TL). Recently, the telomere shortest length assay (TeSLA) was developed to detect critically short telomeres and measure absolute telomeres. Using TeSLA with the internally labeled biotin probe, we measured the TL of bone marrow (BM) aspirates from 52 patients with myelodysplastic syndrome (MDS). A percentage of shortest telomeres (< 1.0 kb (ShTL1.0)) were calculated. ShTL1.0 was correlated to IPSS-R risk (spearman’s rho = 0.35 and *p* = 0.0196), and ShTL1.0 and BM blast (2.61% in < 5% blast, 4.15% in 5–10% blast, and 6.80% in 10–20% blast, respectively, *p* = 0.0332). Interestingly, MDS patients with a shortest TL ≥ 0.787 kb at the time of diagnosis showed better overall survival (OS) and progression-free survival (PFS) than patients with a shortest TL < 0.787 kb in the multivariate analyses (HR = 0.13 and 0.30, *p* = 0.011 and 0.048 for OS and PFS, respectively). Our results clearly show the presence and abundance of critically short telomeres in MDS patients. These pathologic telomeres are associated with IPSS-R which is a validated prognostic scoring system in MDS. Furthermore, they are independent prognostic factors for OS in MDS patients. Future prospective studies are needed to validate our results.

**Supplementary Information:**

The online version contains supplementary material available at 10.1186/s40364-022-00426-9.

## Highlights

Telomere length (TL) has been reported to be important in myelodysplastic syndrome (MDS).A novel TeSLA method demonstrated the presence and abundance of extremely short telomeres (<1.0kb) in MDS.Critically short TL rather than an average TL is associated with the IPSS-R and BM blast in MDS.The shortest TL is an independent prognostic factor for PFS and OS.Short TL should be incorporated into the risk scoring system in MDS in the future.

**Supplementary Information:**

The online version contains supplementary material available at 10.1186/s40364-022-00426-9.

To the editor,

Myelodysplastic syndrome (MDS) is a clonal disorder of hematopoietic stem cell with maturation defect resulting in ineffective hematopoiesis. A recent mouse study showed that dysfunction of telomere drives MDS [1], which indicates that short telomeres might contribute pathogenesis of MDS. When telomeres become extremely short and sufficient loading of the shelterin complex is not possible, cells undergo senescence and induce cell cycle arrest [2].

Several studies have shown that patients with MDS have a shorter telomere length (TL) than healthy controls. However, these studies used conventional telomere restriction fragment (TRF) [3] and interphase quantitative fluorescence in situ hybridization (Q-FISH) [4] method, which provide average TL or relative TL. Several methods to measure the absolute length of short telomere such as STELA (Single telomere length analysis) [5], Universal-STELA [6], and TeSLA (Telomere shortest length assay) [7] have been developed. The most recent method to measure short TL, TeSLA was designed to detect TL up to 18 kb of whole chromosomes [7]. Furthermore, Telomeres below 1.6 kb cannot be visualized by other methods except TeSLA [7].

Here, we investigated TL profile including the proportion of cells with critically short TL in bone marrow (BM) nucleated cells from 52 MDS patients by modified TeSLA with the internally labeled biotin probe. To assess critically short TL as a potential prognostic factor, the association of short telomeres with prognostic risk and clinical outcomes was evaulated (Supplementary materials and methods).

**Fig. 1:**
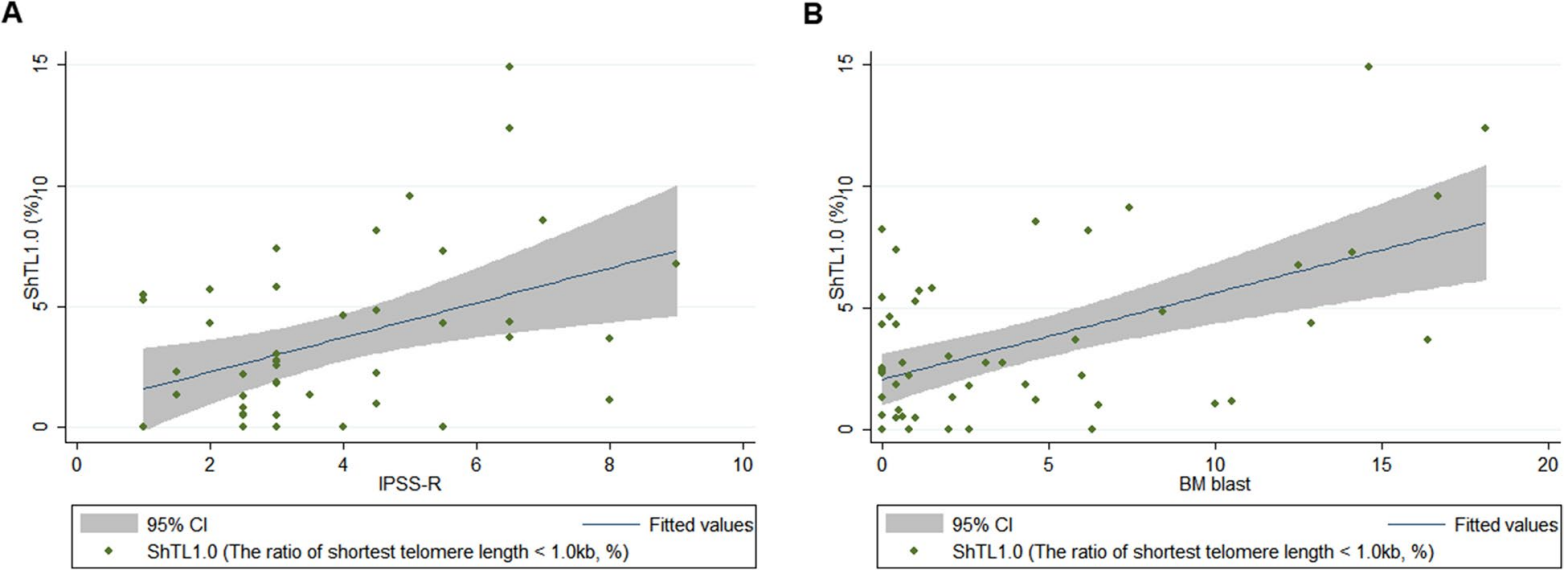
**A** Correlation plot of the ratio of the shortest telomere length below 1.0 kb (%, y axis) and IPSS-R score (x axis). Spearman’s rho = 0.35 and *p* = 0.0196. **B** correlation plot of the ratio of the shortest telomere length below 1.0 kb (%, y axis) and BM blast % (x axis). Spearman’s rho = 0.32 and *p* = 0.0224

**Fig. 2:**
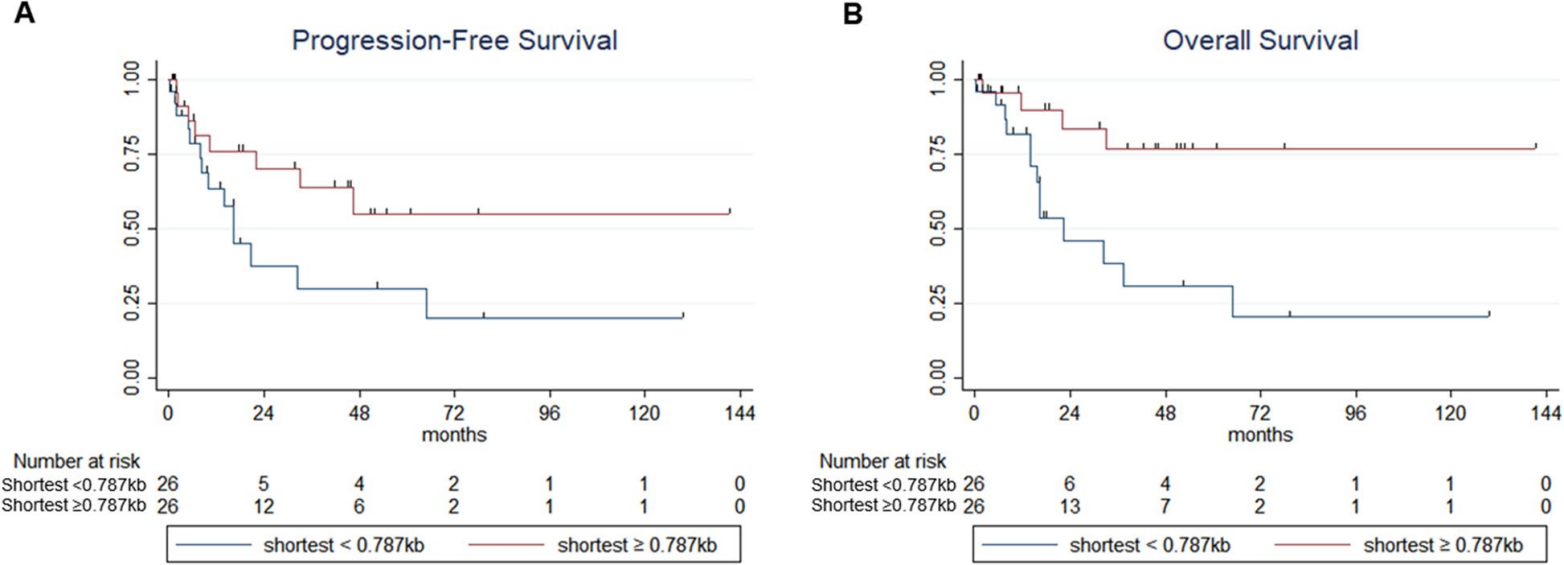
**A** Progression-free survival (PFS) in MDS patients with shortest TL < 0.787 kb vs. with shortest TL ≥ 0.787 kb (median PFS = 16.3 months vs. not reached [NR], *p* = 0.0577) and **B** Overall survival in MDS patients with shortest TL < 0.787 kb vs. with shortest TL ≥ 0.787 kb (median OS = 22.43 months, vs. NR, *p* = 0.0056)

In this study, a total of 52 patients were included. Further characteristics of patients are described in Supplementary Table 2. A median 177 telomere bands (range 75 – 262) were measured for each single patient’s sample. The average TL was 3.18 kb (range, 2.2 – 4.34), which was remarkably shorted than those of healthy populations (5 – 15 kb) previously reported [8, 9]. The shortest TL was 0.787 kb (Supplementary table 3). Our results clearly demonstrated the profiles of shortest telomeres in MDS considering the fact that TL below 1.6 kb was not visualized by other methods except TeSLA [7]. Further details of TL are described in supplementary Table 3. The average TL was not different among MDS (3.32 kb in MDS-SLD, 3.10 kb in MDS-RS-SLD, 3.30 kb in MDS-RS-MLD, 3.18 kb in MDS-MLD, 3.16 kb in MDS-EB1, 3.02 kb in MDS-EB2, 3.43 kb in MDS-U, *p* = 0.71). In contrast, the shortest TL and the ratio of shortest telomere below 1.0 kb (ShTL1.0) showed trends towards positive correlations to MDS subtypes. ShTL1.0 was increased in higher risk MDS group (2.39% in lower risk MDS group and 5.20% in higher risk MDS group, *p* = 0.0227). The scores of IPSS-R and ShTL1.0 was linearly correlated (spearman’s rho = 0.35 and *p* = 0.0196) (Fig. 1A). ShTL1.0 showed a positive correlation with blast percentage in BM (spearman’s rho = 0.32 and *p* = 0.0224) (Fig. 1B). Although ShTL1.0 s according to the presence of thrombocytopenia showed a trend toward a significant difference, we could not find differences of ShTL1.0 values according to the presence of cytopenia or dysplasia (Supplementary Fig. 3). We further investigated if coefficient of variation (CV) of TL was correlated with IPSS-R or BM blasts. The CV of the TL was not significantly correlated with either (Supplementary Fig. 4). MDS patients with a shortest TL ≥ 0.787 kb at the time of diagnosis showed better OS and a trend toward better PFS compared to patients with a shortest TL < 0.787 kb in univariate analyses (HR = 0.23 and 0.44, *p* = 0.011 and 0.064, respectively) (Fig. 2). Multivariate analyses showed that the shortest TL was an independent prognostic factor for PFS and OS both (Supplementary tables 4 and 5). Interestingly, when we analyzed the impact of age on OS according to the cut-off of 60 years, survival difference was consistent in a subgroup of elderly patients (≥ 60 years, *p* = 0.028), showed trends towards differences according to the shortest TL for PFS in young patients (< 60 years, *p* = 0.099) and OS in elderly patients (≥ 60 years, *p* = 0.084) (Supplementary Fig. 5).

Our results suggest that cells with critically short telomeres expand during progression of MDS. The proportion of cells with critically short TL in MDS was a robust indicator to predict poor OS. Our study has a few limitations from its retrospective nature and a small number of patients enrolled into this study. Further, analyses of germline mutations of TERT [10] was not performed in this study. However, our findings on the importance of the presence and abundance of extremely short telomere in the prognosis of MDS can be incorporated into the current prognostic scoring system of the IPSS-R. A large prospective study is needed to establish a new prognostic scoring system in which TL parameters are incorporated as prognostic factors.

## Supplementary Information

**Additional file 1.**

## Data Availability

Data is available upon the request to corresponding author (Dong-Soon Lee, e-mail to soonlee@snu.ac.kr).
