# Supplementary material for: The importance of critically short telomere in myelodysplastic syndrome
Source: Biomark Res. 2022 Nov 10;10:79. doi: 10.1186/s40364-022-00426-9 (PMC9650883; doi:10.1186/s40364-022-00426-9)
Supplement: Supplementary file 1 — Additional file 1. [file 40364_2022_426_MOESM1_ESM.docx]

**Supplementary materials**

**Supplementary Materials and Methods**

Patients

Patients diagnosed as MDS in Seoul National University Hospital from April 2010 to April 2018 were included in this study. All the TL measurements were conducted using BM aspirates of patients obtained with informed consents at the time of diagnosis. This study was approved by the institutional review board of Seoul National University Hospital (2006-179-1135). 2016 World Health Organization (WHO) classification and Revised International Prognostic Scoring System (IPSS-R) were applied for the diagnosis and risk stratification of MDS patients.[1] Cytogenetics was assessed with G-banding results.

Cytopenias were defined using the following definition (hemoglobin <10 g/dL, platelet count <100 × ${10}^{9}$/L, and absolute neutrophil count <1.8 × ${10}^{9}$/L). Higher-risk MDS and lower-risk MDS were classified according to the IPSS-R score of ≤3.5 as lower-risk and >3.5 as higher-risk.[2]

Development of internally labeled biotin probe

TeSLA was performed as described in the original article[3] except for the change in the probe. The internally labeled biotin probe was used instead (supplementary figure 2A).

When designing the biotin with the high sensitivity as the hypersensitive DIG probe, the best bet was to space 22 nucleotides per biotin molecule on the sequence of ‘TG’.[4] Fortunately, the biotin probe was proved highly sensitive as the hypersensitive DIG probe (Supplementary figure 2B). It is known that the binding affinity of biotin and streptavidin is the strongest non-covalent bonds, and the size of streptavidin is smaller than the anti-DIG antibody. Regarding the size of streptavidin and the anti-DIG antibody, streptavidin (52.8 kDa) is approximate ~3 folds smaller than the anti-DIG antibody (IgG, ~150 kDa). It is speculated that inserting more than two biotin-dT is possible to obtain higher sensitivity, which will detect even more of short telomeres below 1.6 kb. The internally labeled biotin probe is commercially available through oligonucleotide suppliers. In addition, commonly used DIG easy Hyb granule (Roche), DIG blocking solution (Roche) and DIG washing buffer (Roche) were compatible with biotin probe and streptoavidin – AP conjugate.

All the oligonucleotides used in TeSLA is described in supplementary table 1.

TeSLA

Genomic DNA was extracted using QIAamps DNA mini kit (Qiagen). Concentration and purity of DNA were measured by Nanodrop 2000 (Thermo Scientific), and integrity was assessed by 20 ng of genomic DNA. Only the genomic DNA samples that met OD 260/280 nm ratio from 1.6 to 2.0 and had an intact band were selected as described in the previous method.[5]

40 µM of adaptor AT and TA mixture were prepared in advance as follows. 40µl of 100 µM adaptor AT were mixed with 40 µl of 100 µM adaptor short oligonucleotide in 1× TSE buffer (10 mM Tris pH 8.0, 50 mM NaCl, 1 mM EDTA) at a final volume of 100 µl. The mixture was incubated at 95 ℃ for 5 min and gradually cooled down to room temperature. The same procedure was performed on the adaptor TA.

50 ng of genomic DNA was ligated with 0.001 µM of TeSLA T1-6 oligos by adding 1000 units of T4 DNA ligase (Enzynomics) and 1 mM of ATP (Enzynomics) to make 20 µl in 1× CutSmart buffer (New England Biolabs). The mixture was incubated at 35 ℃ for 16 h and heat-inactivated at 65 ℃ for 10 min. T1-6 oligos ligated genomic DNA was digested at the sub-telomere regions by restriction enzyme serially. First, two units of CviAII (New England Biolabs) was added to make 30 µl in 1× CutSmart buffer and incubated at 25 ℃ for 2 h. After the CviAII digestion, two units of BfaI (New England Biolabs), MseI (Enzynomics) and NdeI (Enzynomics) were added to make 40 µl in 1× CutSmart buffer and incubated at 37 ℃ for 2 h. Restriction enzyme digested mixture was dephosphorylated by adding one unit of Shrimp Alkaline Phosphatase (New England Biolabs) to make 50 µl in 1× CutSmart buffer and incubated at 37 ℃ for 1 h and heat-inactivated at 80 ℃ for 20 min. 10 µl of the heat-inactivated mixture was ligated with 1 µM of TeSLA adaptor AT and TA mixture by adding 1000 units of T4 DNA ligase and 1 mM of ATP to a final volume of 20 µl in 1× CutSmart buffer. The ligated mixture was incubated at 16 ℃ for 16 h and heat-inactivated at 65 ℃ for 10 min.

PCR was performed by adding 30 pg of DNA, 0.25 µM for each AP, TP primers, and 2.5 unit of Failsafe enzyme (Epicentre) to a final volume of 25 µl in 1× Failsafe premix H (Epicentre) and reacted on the following condition. Initial melt at 94 ℃ for 2 min followed by 26 cycles of 94 ℃ for 15 sec, 60 ℃ for 30 sec, and 72 ℃ for 15 min, with an additional 1 cycle of final extension at 72 ℃ for 15 min.

Amplified DNA was run electrophoresis at 1.5 V/cm for 21 h on 0.85% agarose gel and detected by Southern blot analysis. DNA was transferred to a positively charged nylon membrane (Roche) and UV cross-linked. The cross-linked membrane was incubated in DIG Easy Hyb granule solution (Roche) at 42 ℃ for 2 h in a hybridization incubator and treated 5 nM of the internally labeled biotin probe in DIG Easy Hyb granule solution overnight at 42 ℃. Hybridized DNA was washed with wash buffer1 (2× SSC, 0.1% SDS) at RT for 15 min and twice with prewarmed wash buffer2 (0.5× SSC, 0.1% SDS) at 55℃ for 15 min each, then treated with DIG wash buffer (Roche) briefly at RT for 5 min. The membrane was blocked with 1× DIG blocking solution (Roche) for 1h and incubated with Streptoavidin – AP conjugated antibody (Roche) diluted in 1× DIG blocking solution (0.75:10000) for 30 min at RT. After antibody binding, the membrane was washed twice with DIG wash buffer for 15min each and incubated with 1× DIG detection buffer (Roche) for 5 min at RT for chemiluminescence. Subsequently, telomere bands were detected with CDP-Star (Roche) and visualized with ChemiDOC (Bio-Rad). Blot images were analyzed using software attached in the original article of TeSLA (TeSLAQuant) and average TL, the shortest 20% telomere threshold and the ratio of shortest telomere below 1.6 kb were automatically calculated (Supplementary Figure 1). HeLa cell was analyzed as a positive control of having long telomere above 9 kb.

Statistical methods

All statistical analyses were performed using STATA version 13 (Stata Corp., College Station, TX, USA). Descriptive statistics of mean, standard deviation, and quartile range of TL in each sample were calculated and used in the final analyses. Parameters of the shortest TL and the ratio of the shortest TL below 1.0 kb (ShTL1.0) were also calculated for each patient’s sample and put for further investigations on the meaning of shortest telomeres in MDS in a clinical context. Comparing parameters of TL with clinical characteristics of MDS patients were performed with the Kruskal-Wallis test and the Wilcoxon rank-sum test. Spearman’s rho was used for correlation analysis. Overall survival (OS) was defined as the time frame from the date of the initial diagnosis of MDS to the date of death of any cause or the last follow-up. Progression-free survival (PFS) was defined as the time from the initial diagnosis to a disease progression defined by international working group 2006[6] or death of any cause. Median follow-up time was determined using the Kaplan-Meier method as described previously.[7] Cox regression analysis was performed to investigate prognostic factors for PFS and OS with other clinical variables. Kaplan-Meier method were used for survival estimation and compared using the log-rank test. Two-sided p < 0.05 was considered significant.

**Supplementary Figures**

Supplementary Figure 1.


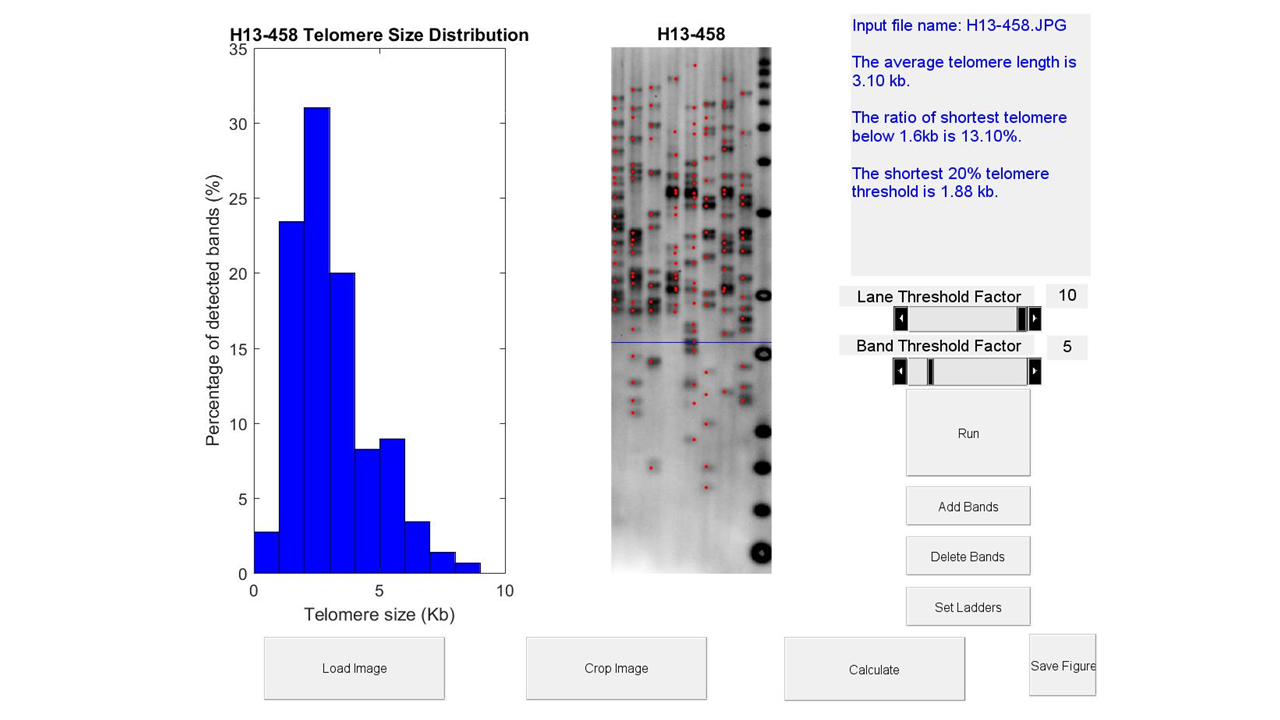


Detection procedure of TeSLA blot using TeSLAQuant software. Telomere bands are detected using TeSLAQuant software attached in the original article of TeSLA. Telomere bands are automatically detected and marked as dots. The distribution of telomere bands is displayed as a density plot. Average TL, the shortest 20% of telomere threshold and the ratio of shortest telomere below 1.6 kb are calculated as described in the box below the density plot (1.6 kb criteria can be changed to 1.0 kb to calculate the ratio of the shortest telomere below 1.0 kb, data not shown).

Supplementary Figure 2.

The design of the internally labeled biotin probe and dot blot assay. (A) Two biotin-dTs are inserted and spaced 22 nucleotides. (B) Dot blot assay evaluating the sensitivity of the internally labeled biotin probe. Serially diluted probes (100 fmol to 0.2 fmol) and distilled water were loaded. Detection was performed, as described in the dot blot assay. The assay was conducted in triplicate.

Supplementary figure 3.


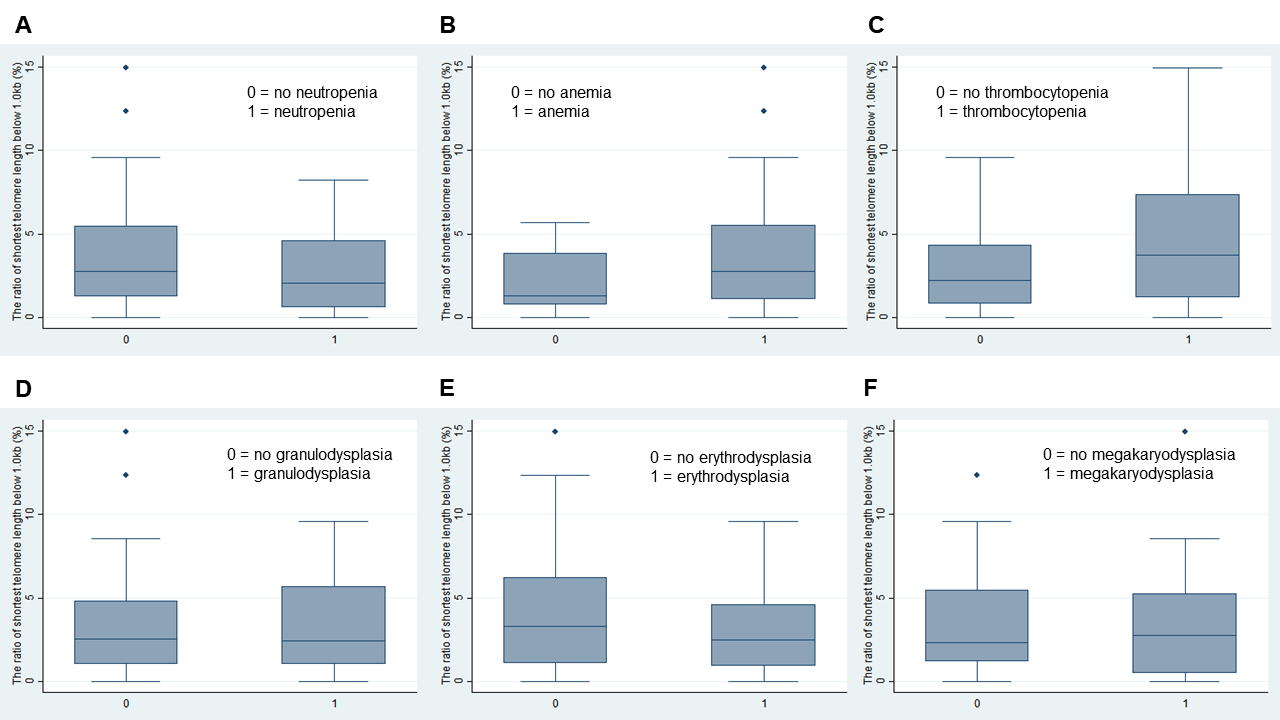


ShTL1.0 according to the cytopenia in peripheral blood and dysplasia in bone marrow. (A) shTL1.0 according to the presence of neutropenia (ANC <1000/㎕), shTL1.0 = 2.71% in no thrombocytopenia vs. 4.59% in thrombocytopenia, p = 0.0846, (B) shTL1.0 = 2.18% in no anemia vs. 3.79% in anemia (Hb <10.0g/dL), p = 0.23, (C) shTL1.0 = 2.71% in no thrombocytopenia vs. 4.59% in thrombocytopenia (platelet < 100,000/㎕), p = 0.0846, (D) shTL1.0 = 3.66% in no granulodysplasia vs. 3.38% in granulodysplasia, p = 0.92, (E) shTL1.0 = 4.39% in no erythrodysplasia vs. 3.17% in no erythrodysplasia, p = 0.45, (F), shTL1.0 = 3.43% in no megakaryodysplasia vs. 3.82% in megakaryodysplasia, p = 0.93.

Supplementary figure 4


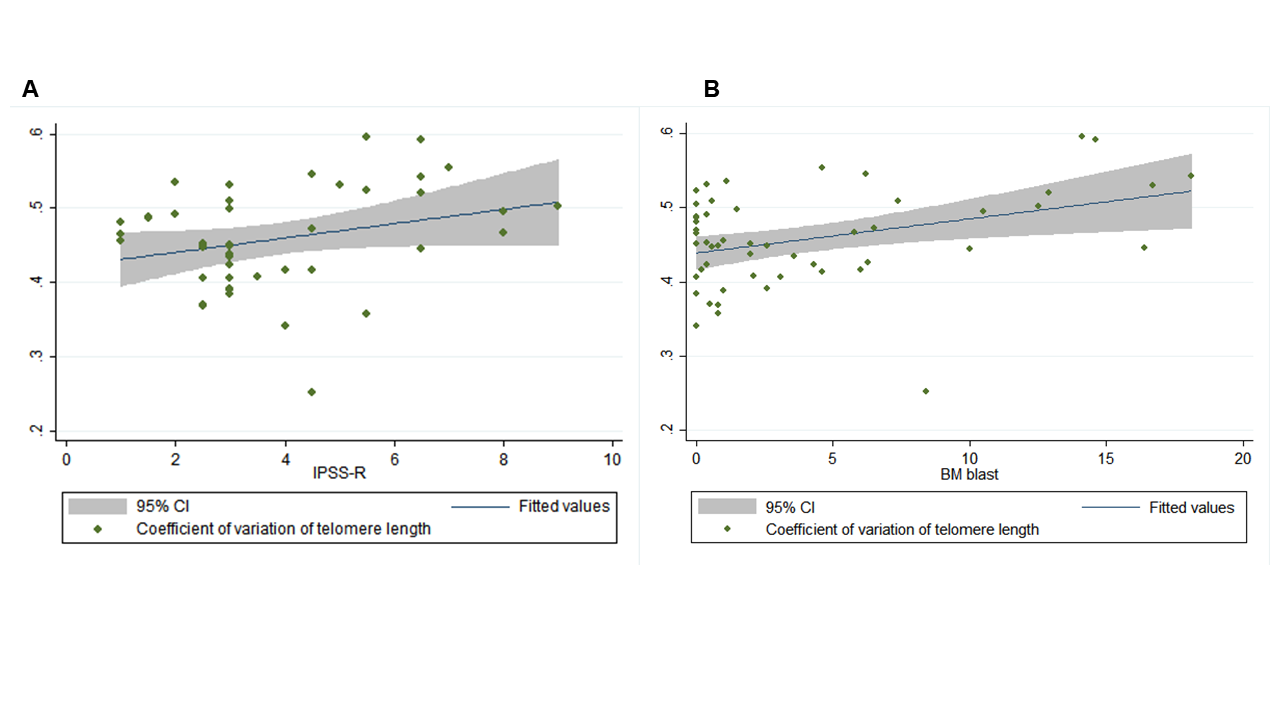


Coefficient of variation (standard deviation / average) was not significantly correlated with (A) IPSS-R score (Spearman’s rho = 0.242, p = 0.114) and (B) BM blast % (Spearman’s rho = 0.229, p = 0.103).

Supplementary figure 5


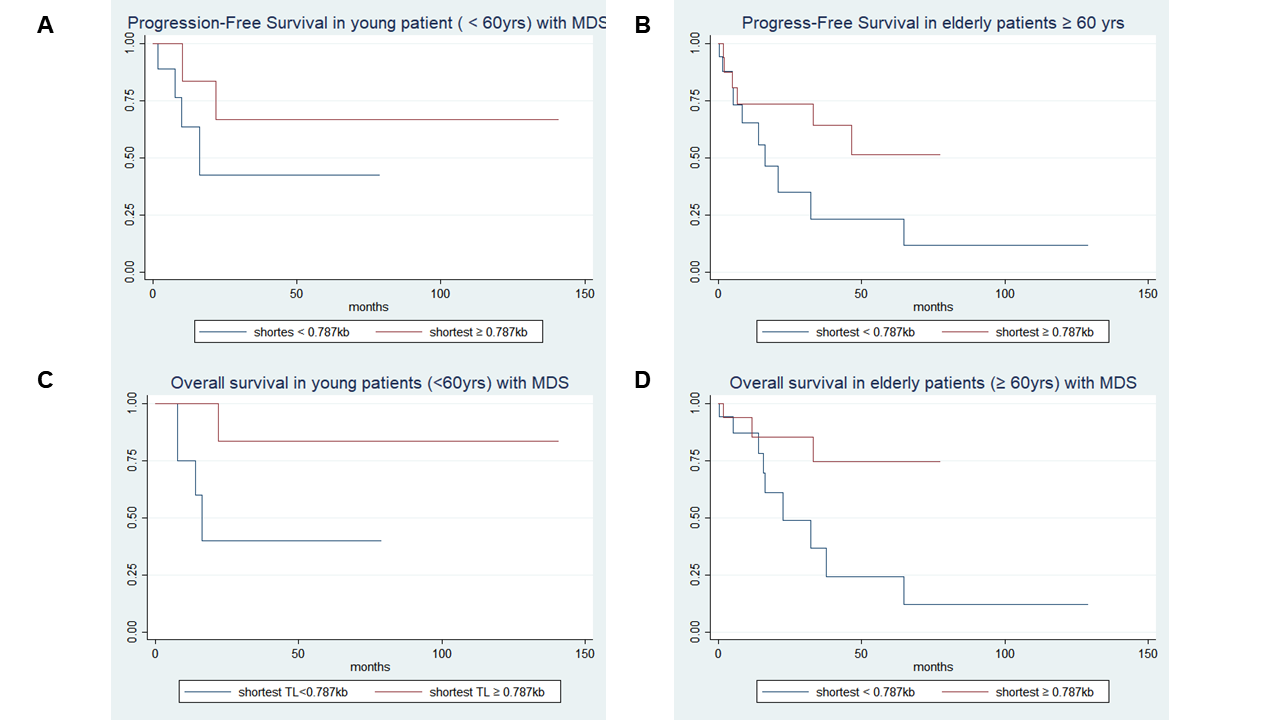


Progression-free survival (PFS) and overall survival (OS) according to age group ( < 60yrs vs. ≥ 60 yrs). PFS was not significantly different in young age group (p = 0.30), showed trends towards significant differences (p = 0.099 for PFS in elderly group and p = 0.084 for OS in young age group), while OS in elderly group was different according to the length of shortest telomere (p = 0.028).

**Supplementary tables**

| Supplementary table 1. The sequence of oligonucleotides used for TeSLA | |
| --- | --- |
| Oligos | Sequence |
| TeSLA-T1 | 5′-ACT GGC CAC GTG TTT TGA TCG ACC CTA AC-3′ |
| TeSLA-T2 | 5′-ACT GGC CAC GTG TTT TGA TCG ATA ACC CT-3′ |
| TeSLA-T3 | 5′-ACT GGC CAC GTG TTT TGA TCG ACC TAA CC-3′ |
| TeSLA-T4 | 5′-ACT GGC CAC GTG TTT TGA TCG ACT AAC CC-3′ |
| TeSLA-T5 | 5′-ACT GGC CAC GTG TTT TGA TCG AAA CCC TA-3′ |
| TeSLA-T6 | 5′-ACT GGC CAC GTG TTT TGA TCG AAC CCT AA-3′ |
| TeSLA adaptor short | 5′-GGT TAC TTT GTA AGC CTG TC [SpcC3]-3′ |
| TeSLA adaptor AT | 5′-[Phos] TAG ACA GGC TTA CAA AGT AAC CAT GGT TA GGA GAA TTC TGT CGT CTT CAC GCT ACA TT [SpcC3]-3′ |
| TeSLA adaptor TA | 5′-[Phos] ATG ACA GGC TTA CAA AGT AAC CAT GGT AT GGA GAA TTC TGT CGT CTT CAC GCT ACA TT [SpcC3]-3′ |
| AP | 5′-TGT AGC GTG AAG ACG ACA GAA-3′ |
| TeSLA-TP | 5′-TGG CCA CGT GTT TTG ATC GA-3′ |
| [Phos]: 5′ phosphorylation; [SpcC3]: C3 spacer | |

| Supplementary table 2. Baseline characteristics | |
| --- | --- |
| Characteristics | (n = 52) |
| Age (years) | 66 [15-88] |
| Sex |  |
| Male | 37 (71.1%) |
| Female | 15 (28.9%) |
| Subtype |  |
| MDS-SLD | 6 (11.5%) |
| MDS-MLD | 16 (30.8%) |
| MDS-RS-SLD | 4 (7.7%) |
| MDS-RS-MLD | 4 (7.7%) |
| MDS-EB1 | 8 (15.4%) |
| MDS-EB2 | 9 (17.3%) |
| MDS-U | 5 (9.6%) |
| IPSS-R (n = 44)* |  |
| Very Low | 5 (11.4%) |
| Low | 20 (45.5%) |
| Intermediate | 7 (15.9%) |
| High | 6 (13.6%) |
| Very High | 6 (13.6%) |
| Number of cytopenias** |  |
| 0 | 3 (5.8%) |
| 1 | 23 (44.2%) |
| 2 | 16 (30.8%) |
| 3 | 10 (19.2%) |
| Presence of dysplasia |  |
| Erythrodysplasia | 35 (67.3%) |
| Granulodysplasia | 20 (38.5%) |
| Megakaryodysplasia | 14 (26.9%) |
| Complete blood count (Average) |  |
| White blood cell (/μl) | 3265 [800 – 77780] |
| Hemoglobin (g/dL) | 8.4 [4.4 - 13.2] |
| Platelet count (×10³/μl) | 110 [3 - 443] |
| Absolute neutrophil count (/μl) | 1652 [150 - 53668] |
| BM Blast (n = 46)*** |  |
| ≤2% | 25 (54.4%) |
| >2 ~ <5% | 7 (15.2%) |
| 5-10% | 6 (13.0%) |
| >10% | 8 (17.4%) |
| Karyotype (n = 50)**** |  |
| Normal | 19 (37.3%) |
| Abnormal (1~3) | 23 (45.1%) |
| Complex (>3) | 9 (17.7%) |
| MDS, Myelodysplastic syndrome; MDS-SLD, MDS with single lineage dysplasia; MDS-MLD, MDS with multilineage dysplasia; MDS-RS, MDS with ring sideroblasts; MDS-RS-SLD, MDS-RS with single lineage dysplasia; MDS-RS-MLD, MDS-RS with multilineage dysplasia; MDS-EB, MDS with excess blasts; MDS-U, MDS, unclassifiable; IPSS-R, Revised international prognostic score system.  * 8 samples were excluded due to the poor quality of BM aspirates (n=6) and the absence of G-banding result (n=2)  ** Hb <10, Plt <100k, ANC <1000 *** 46 samples were analyzed in the BM blast percentage result  (6 samples were excluded for their poor quality of BM aspirates) | |

*** G-banding result was not available in 2 samples

Supplementary Table 3. Telomere length parameters

| Average Telomere length in each patient (kb) [range] |  |
| --- | --- |
| median [range] | 3.18 [2.2 – 4.34] |
| Standard deviation of telomere length | 1.426 [0.983 – 1.948] |
| Coefficient variation of telomere length | 0.452 [0.252 – 0.595] |
| The shortest of telomere threshold (kb) | 0.787 [0.441 – 1.356] |
| The shortest 5% of telomere threshold (kb) | 1.216 [0.81 – 1.757] |
| The shortest 10% of telomere threshold (kb) | 1.459 [0.914 – 2.242] |
| The shortest 20% of telomere threshold (kb) | 1.90 [1.17 – 2.61] |
| The shortest 25% of telomere threshold (kb) | 2.07 [1.24 – 2.78] |
| Median telomere length (50% quartile) (kb) | 2.98 [1.90 – 4.035] |
| The 75% of telomere threshold (kb) | 4.098 [2.726 – 8.239] |
| The longest telomere length (kb) | 7.246 [5.15 – 9.07] |
| The ratio of shortest telomere below 1.0 kb (%) | 2.48 [0 – 14.925] |
| The ratio of shortest telomere below 1.6 kb (%) | 13.23 [1.35 – 39.3] |
| The ratio of longest telomere above 5.0 kb (%) | 13.38 [0.77 – 34.615] |

Supplementary table 4. Shortest TL and overall survival

|  |  | |  | Univariate | |  | Multivariate | |
| --- | --- | --- | --- | --- | --- | --- | --- | --- |
|  |  | |  | HR (95% CI) | *p* value |  | HR (95% CI) | *p* value |
| Age >60 | |  | | 1.32 (0.47-3.76) | 0.60 |  | 1.34 (0.37-4.91) | 0.66 |
| Sex (male) | | | | 4.08 (0.93-17.91) | 0.062 |  | 2.81 (0.52-15.18) | 0.23 |
| IPSS-R | | Very low (referent) | |  |  |  |  |  |
|  | | Low | | 0.79 (0.12-5.04) | 0.80 |  | 1.84 (0.26-12.85) | 0.54 |
|  | | Intermediate | | 1.87 (0.30-11.55) | 0.50 |  | 3.86 (0.57-25.92) | 0.17 |
|  | | High | | 4.12 (0.68-24.89) | 0.12 |  | 15.28 (1.75-133.13) | 0.014 |
|  | | Very high | | 2.24 (0.29-17.55) | 0.44 |  | 3.48 (0.37-32.29) | 0.27 |
| Shortest TL (>0.787 kb) | | | | 0.23 (0.074-0.71) | 0.011 |  | 0.13 (0.027-0.63) | 0.011 |

Supplementary table 5. Shortest TL and progression-free survival

|  |  | |  | Univariate | |  | Multivariate | |
| --- | --- | --- | --- | --- | --- | --- | --- | --- |
|  |  | |  | HR (95% CI) | *p* value |  | HR (95% CI) | *p* value |
| Age >60 | |  | | 1.50 (0.59-3.85) | 0.40 |  | 2.75 (0.80-9.44) | 0.11 |
| Sex (male) | | | | 1.85 (0.68-5.07) | 0.23 |  | 1.03 (0.29-3.68) | 0.96 |
| IPSS-R | | Very low (referent) | |  |  |  |  |  |
|  | | Low | | 1.00 (0.17 – 5.77) | 0.99 |  | 2.01 (0.30-13.72) | 0.48 |
|  | | Intermediate | | 2.52 (0.45-14.12) | 0.29 |  | 5.17 (0.75-35.44) | 0.094 |
|  | | High | | 3.99 (0.68-23.52) | 0.13 |  | 10.12 (1.34-76.52) | 0.025 |
|  | | Very high | | 5.21 (-.87-31.27) | 0.071 |  | 8.21 (1.05-63.94) | 0.044 |
| Shortest TL (>0.787 kb) | | | | 0.44 (0.18-1.05) | 0.064 |  | 0.30 (0.088-0.99) | 0.048 |

**References**

1. Greenberg PL, Tuechler H, Schanz J, Sanz G, Garcia-Manero G, Sole F, et al. Revised international prognostic scoring system for myelodysplastic syndromes. Blood. 2012;120(12):2454-65.

2. Giagounidis A. Current treatment algorithm for the management of lower-risk MDS. Hematology Am Soc Hematol Educ Program. 2017;2017(1):453-459.

3. Lai TP, Zhang N, Noh J, Mender I, Tedone E, Huang E, et al. A method for measuring the distribution of the shortest telomeres in cells and tissues. Nat Commun. 2017;8(1):1356.

4. Lai TP, Wright WE, Shay JW. Generation of digoxigenin-incorporated probes to enhance DNA detection sensitivity. Biotechniques. 2016;60(6):306-9.

5. Kimura M, Stone RC, Hunt SC, Skurnick J, Lu X, Cao X, et al. Measurement of telomere length by the Southern blot analysis of terminal restriction fragment lengths. Nat Protoc. 2010;5(9):1596-607.

6. Cheson BD, Greenberg PL, Bennett JM, Lowenberg B, Wijermans PW, Nimer SD, et al. Clinical application and proposal for modification of the International Working Group (IWG) response criteria in myelodysplasia. Blood. 2006;108(2):419-25.

7. Schemper M, Smith TL. A note on quantifying follow-up in studies of failure time. Control Clin Trials. 1996;17(4):343-6.
